# Neutrophil Counts in Healthy South African Infants: Implications for Enrollment and Adverse Event Grading in Clinical Trials in an African Setting

**DOI:** 10.1016/j.ympdx.2019.100005

**Published:** 2019-07-18

**Authors:** Anthonet Koen, Lisa Jose, Shabir A. Madhi, Alan Fix, Stanley Cryz, Michelle J. Groome

**Affiliations:** 1 Medical Research Council: Respiratory and Meningeal Pathogens Research Unit, Faculty of Health Sciences, University of the Witwatersrand, Johannesburg, South Africa; 2 Department of Science and Technology/National Research Foundation: Vaccine Preventable Diseases, University of the Witwatersrand, Johannesburg, South Africa; 3 PATH, Washington, DC

## Abstract

Absolute neutrophil counts are used to assess eligibility and safety during clinical trials but the toxicity grading scale used can affect enrollment and reporting of adverse events. During a trial investigating a parenteral rotavirus vaccine in South Africa, we excluded otherwise healthy infants without HIV infection from participation owing to neutropenia.

**Trial registration:**

ClinicalTrials.gov: NCT02109484.

Safety evaluation is a critical component of clinical trials in the early stages of drug or vaccine development. Hematological values are used, both for assessment of eligibility and grading of adverse events, and the absolute neutrophil count (ANC) is commonly measured before and after the intervention.^1^ Although important to accurately assess safety, misclassification of adverse events could undermine enrollment into studies, as well as exaggerate the frequency of adverse event reporting. ANC are subject to age, sex, and geoethnic variations.^2^ Both adults and children of African and Middle Eastern descent may have lower white cells counts, including ANC, compared with those of European descent.^3^, ^4^ Toxicity grading is often based on values derived from populations with different ethnic, racial, geographic, and socioeconomic backgrounds from that of the study population, which may lead to the application of a scale not relevant for safety assessment in that population. Thus, consideration of the grading scale used to assess hematological values is important, because this factor may affect the eligibility and interpretation of potential safety signals during clinical trials.

During the conduct of a clinical trial investigating a parenteral rotavirus vaccine in South Africa, we excluded otherwise healthy infants without HIV infection from participation owing to abnormal ANC when using a grading table provided by the local laboratory performing the hematological safety testing. This resulted in revision of the grading table used during the remainder of the study. We assessed the impact that the choice of grading scale had on enrollment and the reporting of adverse events in our study.

## Methods

A double-blind, randomized, placebo-controlled, dose escalation study investigating the safety and immunogenicity of a parenteral subunit monovalent rotavirus vaccine (P2-VP8-P[8]) was conducted at the Respiratory and Meningeal Pathogens Research Unit, Soweto, South Africa, from March 2014 to October 2015 (ClinicalTrials.gov: NCT02109484). A description of the study methods, and safety and immunogenicity results of the study have been published.^5^ Healthy, term infants without HIV infection (aged ≥6 and <8 weeks) were screened for eligibility 1 to 7 days before enrollment. Eligibility was determined by medical history, clinical examination, screening laboratory tests, and fulfilment of all the inclusion and absence of any exclusion criteria.^5^ Laboratory testing included a white blood cell count with 5-part differential, platelet count, total bilirubin, albumin, creatinine, and alanine transaminase were tested at screening and 7 days after the first injection of vaccine or placebo. Blood samples were collected at the research site, kept at ambient temperature and transported to a South African National Accreditation System-accredited local laboratory, Clinical Laboratory Services, Johannesburg, South Africa, on the day of collection. Blood analyses were performed in real time using standardized methods, including an automated full blood count and white cell differential analysis performed using a Beckman Coulter LH-750 Haematology Analyser (Beckman Coulter, Pasadena, California). Results were reviewed promptly by the principal investigator or designee and abnormal results were graded from mild (grade 1) to life threatening (grade 4) according to a non-age-specific toxicity grading table provided by the laboratory. Grade 1 laboratory abnormalities were not considered to be exclusionary at screening, unless judged to be clinically significant by the principal investigator. Infants with abnormal values categorized as grade 2 or above were considered screening failures.

The initial ANC grading was based on a hematology grading table provided by Clinical Laboratory Services. During the course of the study, a high prevalence of mild, moderate, or severe neutropenia (grades 1, 2, or 3) was noted among clinically healthy infants during screening. This prompted the study Safety Review Committee (SRC) to review the grading table for assessing ANC which was in use at that time. Possible, commonly-used alternative grading scales (Table) were reviewed by the SRC, who recommended revision of the ANC grading to reflect the Division of AIDS (DAIDS) Table for Grading the Severity of Adult and Pediatric Adverse Events, Version 1.0, updated August 2009.^6^

**Table:** Comparison of commonly used toxicity grading scales/tables for assessing ANC (cells/mm^3^)

Toxicity grading scales	Age group	Mild (grade 1)	Moderate (grade 2)	Severe (grade 3)	Life threatening (grade 4)
P2-VP8 study - initial	≥2 and <3 years	1500-2000	900-1499	<900	<250
P2-VP8 study - revised	≥6 and <8 weeks	1000-1300	750-999	500-749	<500
CLS	Not specified	1500-2000	900-1499	<900	–
DAIDS V1.0	>7 days	1000-1300	750-999	500-749	<500
DAIDS V2.0/V2.1	>7 days	800-1000	600-799	400-599	<400
DMID	7-60 days	1200-1800	900-1199	500-899	<500
DMID	>60 days	750-1200	400-749	250-399	<250
FDA	Healthy adult and adolescent	1500-2000	1000-1499	500-999	<500
CTCAE 5.0	Not specified	1500-<LLN	1000-1499	500-999	<500

*CLS*, Clinical Laboratory Services, Johannesburg, South Africa; *CTCAE*, Common Terminology Criteria for Adverse Events Version 5.0 Nov 2017 (https://ctep.cancer.gov/protocolDevelopment/electronic_applications/docs/CTCAE_v5_Quick_Reference_8.5x11.pdf); *DAIDS*, DAIDS Table for Grading the Severity of Adult and Pediatric Adverse Events; V1.0 Dec 2004, clarification Aug 2009 (https://rsc.niaid.nih.gov/sites/default/files/table-for-grading-severity-of-adult-pediatric-adverse-events.pdf); V2.0 – November 2014 (https://rsc.niaid.nih.gov/sites/default/files/daids-ae-grading-table-v2-nov2014.pdf); V2.1 – July 2017 (https://rsc.niaid.nih.gov/sites/default/files/daidsgradingcorrectedv21.pdf); *DMID*, Division of Microbiology and Infectious Diseases Pediatric Toxicity Tables Nov 2007 (https://www.niaid.nih.gov/sites/default/files/dmidpedtox.pdf); *FDA*, Guidance for Industry, Toxicity Grading Scale for Healthy Adult and Adolescent Volunteers Enrolled in Preventive Vaccine Clinical Trials 2007 (https://www.fda.gov/downloads/BiologicsBloodVaccines/GuidanceComplianceRegulatoryInformation/Guidances/Vaccines/UCM091977.pdf); *LLN*, lower limit of normal.

Proportions were compared using the χ^2^ and Fisher exact tests, as appropriate, and continuous data were compared using the Wilcoxon rank sum test. All tests were 2 sided and *P* < .05 was considered significant. Data analysis was performed using STATA version 13.1 (StataCorp, College Station, Texas). The study was approved by the Human Research Ethics Committee, University of the Witwatersrand; the Medicines Control Council, South Africa; and the Western Institutional Review Board, US. Written informed consent was obtained from a parent before enrollment of the child.

## Results

**Figure 1:**
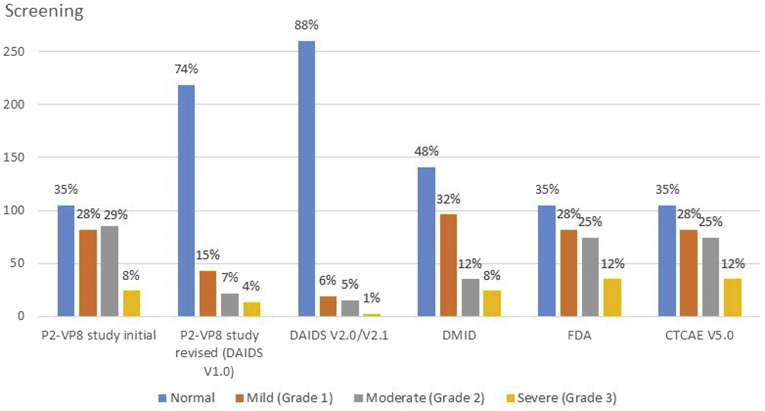
Grading of ANCs in healthy HIV-uninfected South African infants at screening (n = 296) using different toxicity grading scales. *CTCAE*, Common Terminology Criteria for Adverse Events Version 5.0 Nov 2017; *DAIDS*, DAIDS Table for Grading the Severity of Adult and Pediatric Adverse Events; V1.0 Dec 2004, clarification Aug 2009; V2.0 – November 2014; V2.1 – July 2017; *DMID*, Division of Microbiology and Infectious Diseases Pediatric Toxicity Tables Nov 2007; *FDA*, Guidance for Industry, Toxicity Grading Scale for Healthy Adult and Adolescent Volunteers Enrolled in Preventive Vaccine Clinical Trials 2007.

A baseline (screening) laboratory evaluation was available for 296 infants. At screening, the mean age was 43 days (SD ± 4; range, 35-55), mean birth weight was 3162 g (SD ± 419), 136 (46%) were male, 293 (99%) of black race, 35 (12%) were HIV exposed, and all were confirmed to be HIV uninfected. The median ANC was 1780 cells/mm^3^ (IQR, 1285-2365 cells/mm^3^; range, 520-5880 cells/mm^3^). There was no significant difference in median ANC by sex (males, 1780 cells/mm^3^; females, 1765 cells/mm^3^; *P* = .200) or HIV-exposure status (HIV exposed, 1920 cells/mm^3^; HIV-unexposed, 1760 cells/mm^3^; *P* = .174). The initial grading table was used from July 15, 2014, to August 19, 2014. Of 57 infants screened during this period, 14 (25%) were assessed as mild, 12 (21%) as moderate, and 4 (7%) as severely neutropenic; thus, 16 patients (28%) were excluded from enrollment solely owing to moderate or severe neutropenia (ANC of <1500 cells/mm^3^). From August 27, 2014, the revised grading was used to assess ANC decrease and eligibility. For the remainder of the screening period, 239 infants were screened and 37 (15%) were classified as mild, 18 (7%) as moderate, and 11 (5%) as severely neutropenic. Twenty-nine infants (12%) were excluded from participation solely owing to moderate or severe neutropenia when using the revised grading table (ANC of <1000 cells/mm^3^), compared with the 16 (28%) during initial screening (*P* = .003). If the initial grading had been used for the entire study period, 109 of the 296 screened infants (37%) would have been excluded owing to moderate or severe neutropenia, whereas only 35 (12%) would have been excluded had the revised grading (DAIDS Version 1.0) been used from study start. If Version 2.0/2.1 of the DAIDS grading table,^7^ only released after the SRC's deliberation, had been used for our entire study, only 17 (6%) of those screened would have been excluded owing to moderate or severe neutropenia, compared with the 12% noted using the previous version of that table. Grading of ANC at screening using common grading scales is shown in Figure 1.

**Figure 2:**
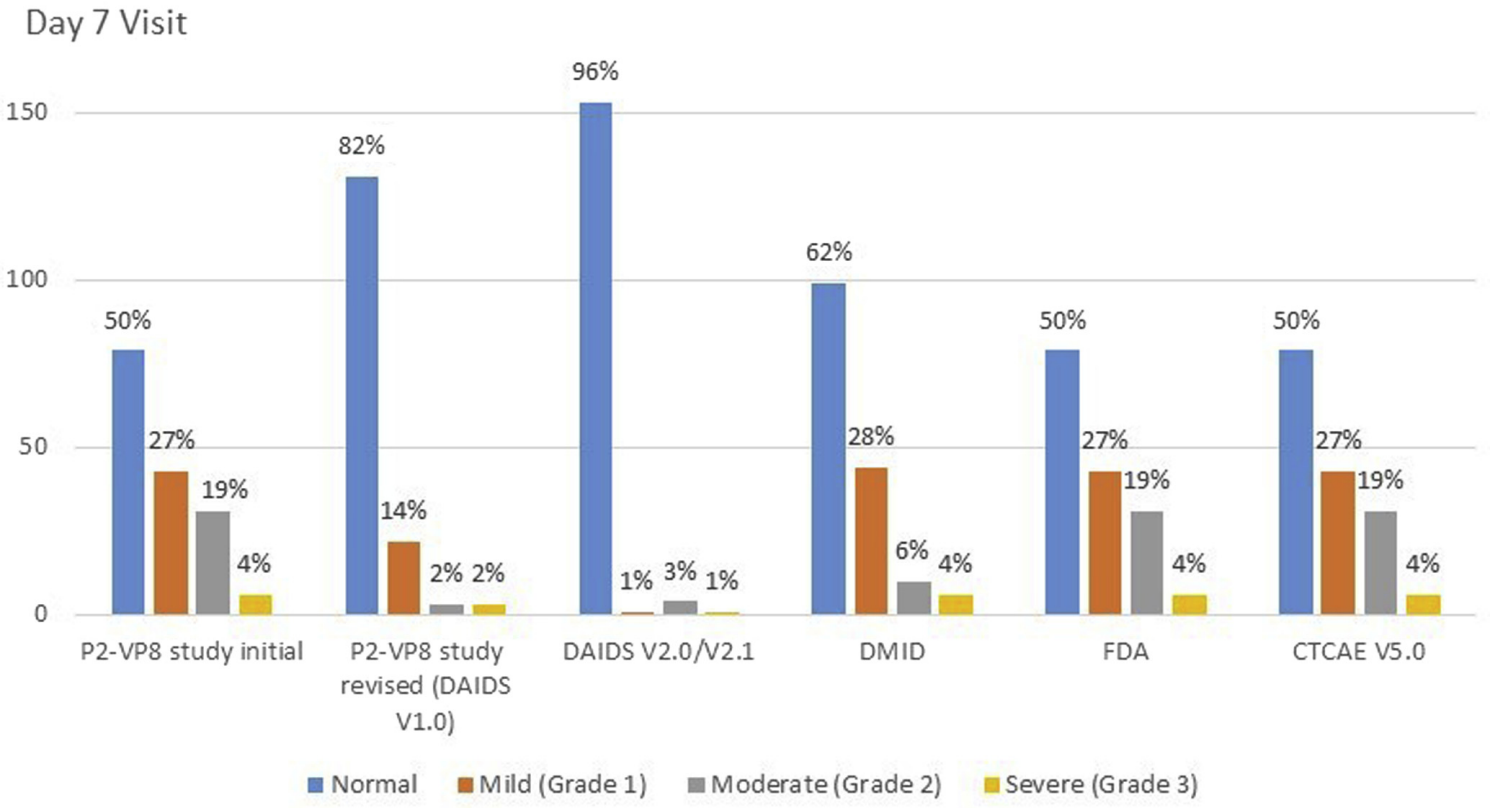
Grading of ANCs in healthy HIV-uninfected South African infants at day 7 after the first vaccination (n = 159) using different toxicity grading scales. There were no infants assessed as having life-threatening neutropenia using any of the grading scales. *CTCAE*, Common Terminology Criteria for Adverse Events Version 5.0 Nov 2017.

One hundred and fifty-nine infants were randomized and had a hematology result available on day 7 after the injection. Two infants were assessed as having severe neutropenia after the injection (ANC of 500 and 620 cells/mm^3^). The revised study grading scale was in use at this time (DAIDS V1.0). This met a study-stopping criterion and triggered a pause in study enrollment and vaccination. Safety data were reviewed by the SRC, who did not find evidence for an association with the study vaccine, and it was recommended that the study continue. Subsequently, 1 other infant was assessed as having severe neutropenia after the injection (ANC of 710 cells/mm^3^). If DAIDS grading table V2.1 had been in use, only one of these infants would have been assessed as severe neutropenia and a study pause would not have occurred. Grading of ANC after the injection using common grading scales is shown in Figure 2. None of these events were assessed as serious adverse events, no clinical adverse events were reported for any of these participants at the time of the decreased ANC, and all episodes of neutropenia resolved spontaneously.

## Discussion

Our study demonstrates how choice of toxicity grading scale in a clinical trial can have significant impact on both enrollment into the study and postintervention safety assessments. Although we attempted to take our setting into account by adopting a grading scale provide by the local laboratory, this resulted in a relatively high exclusion rate among a healthy infant population screened for enrollment, with associated time and cost implications. Use of a revised grading scale for assessing ANC among screened infants (DAIDS V1.0), led to 12% of infants being assessed as having moderate to severe neutropenia, significantly less than the 28% using the initial grading scale. Two infants presented with severe neutropenia (ANC of <750 cells/mm^3^, as per the revised grading scale) after the injection, necessitating a pause in study enrollment and vaccination as a study-halting criterion was met. This resulted in successfully screened infants not being enrolled and additional infants having to be screened once the study resumed, delays in scheduled vaccination of enrolled infants, and an inconvenience to parents because visit dates had to be amended, with further time and cost implications to the study conduct. DAIDS grading table V2.0 was released in November 2014 (Version 2.1 in July 2017), several months after we revised the grading for our study. The new DAIDS version followed an extensive literature search and review of clinical trial data, and the definition of grade 3 decrease in ANC was modified to 400-599 cells/mm^3^.^7^ If this grading had been in use during our study, only 1 infant would have been assessed as severely neutropenic and the study would not have halted, thus, averting the associated impact on study timelines, ethics, and regulatory reporting, rescreening, inconvenience to participants, and costs.

A review of the published literature on neutropenia reported during vaccine trials showed that neutropenia had been reported as an adverse event in >30 phase I and II vaccine trials on PubMed, often being the most commonly reported laboratory adverse event abnormality, as was the case in our study.^1^ Neutropenia was shown to generally be a common, transient, and clinically asymptomatic postvaccination occurrence with a benign clinical outcome and lack of complications associated with the episodes of neutropenia. This was also our experience; all neutropenic infants in our study were clinically well, the neutropenia resolved spontaneously without any complications, and it was not associated with receipt of vaccine vs placebo. The reason for the high prevalence of low neutrophil counts in our population is not entirely clear, but likely reflects normal values for this population, which needs to be elucidated further. Our population consisted of healthy, term infants free of any underlying infections and other pathology, including HIV infection, and not using any medication that could contribute to a decreased ANC except for HIV-exposed uninfected children who were receiving nevirapine for prophylaxis. The use of antiretroviral medication is a known cause of neutropenia, but our results did not show decreased ANC amongst HIV-exposed compared with HIV-unexposed infants.^8^ The most likely explanation for these observed low ANC values would be a benign genetic disposition, described among individuals of African origin, such as our Soweto infant population, which was composed almost exclusively of black Africans.^4^, ^9^, ^10^, ^11^

Our findings were similar to those from other African countries. A Zimbabwean study to establish normal hematological values and assess the prevalence of neutropenia, as defined by the DAIDS V1.0 grading table, showed that 24% of healthy, term, HIV-uninfected, black infants had relative neutropenia of any grade at 6 weeks of age.^12^ The median ANC was 1700 cells/mm^3^, which was very similar to that found in our study as well as a study in Tanzanian children <1 year of age (median, 1700 cells/mm^3^; 95% reference interval, 700-4600).^13^ During a clinical trial investigating a HIV vaccine, a high rate of low ANC was observed, with significant differences in ANC among ethnic/race groups at 24, 76, and 104 weeks of age, and black infants showing the lowest ANC values. Black participants were also more likely to develop neutropenia after vaccination, unrelated to the study arm.^14^ A population study in Uganda reported a median ANC of 2100 cells/mm^3^ (90% CI, 900-4400 cells/mm^3^) in 373 healthy infants <1 year of age.^15^ Our experience was, however, not in keeping with results from a South African pediatric population in the Western Cape, an area with a high frequency of mixed ancestry, where results were more consistent with those reported from European populations. In that study, the median ANC in infants 0-3 months of age was 2360 cells/mm^3^ (95% CI, 1260-5200 cells/mm^3^).^16^ This difference may be due to the proportion of participants of mixed ethnic ancestry in their study, whereas our study population almost exclusively comprised black Africans. Although attempts have been made to establish hematology reference ranges for healthy African adults,^17^ we are not aware of a similar study among children <5 years of age. Literature are lacking partially owing to ethical considerations in obtaining samples from children to generate reference ranges.

Although limited to a single clinical trial, our study clearly demonstrated the variable assessment of moderate to severe neutropenia depending on the grading scale used, and the implications this had on the study. The exclusion of otherwise healthy individuals translates into increased time and cost, and also prevents healthy individuals from participating in clinical trials, making results less generalizable to the healthy population being studied. Further, the misclassification of monitoring laboratory values can lead to unnecessary study disruption. A comprehensive understanding of reference ranges and site-specific factors which may influence study toxicity criteria is required to ensure that healthy participants are not excluded inappropriately. In addition, it is important to carefully consider the choice of toxicity grading scale.
